# Establishment of an Isolation System for Extracellular Vesicles of *Fusarium oxysporum* and Its Proteomic Analysis

**DOI:** 10.3390/jof11120884

**Published:** 2025-12-15

**Authors:** Jiayi Lou, Guangjin Hu, Xuan Wang, Qiang Liu, Yuwei Chen, Weichun Zhao

**Affiliations:** 1 School of Basic Medical Sciences, Zhejiang Chinese Medical University, Hangzhou 310053, China; 2 School of Life Sciences, Zhejiang Chinese Medical University, Hangzhou 310053, China

**Keywords:** *Fusarium oxysporum*, extracellular vesicles, isolation system establishment, proteomics, *Fo*-EVs tagged proteins

## Abstract

Extracellular vesicles (EVs) secreted by *Fusarium oxysporum* play an important role in the process of its infestation of the host, but the in vitro research system for EVs of *F. oxysporum* (*Fo*-EVs) has not yet been improved, and the mechanism of its action remains unclear. In this study, particle size distribution, particle concentration, number of particles per unit of protein, number of particles per unit of mycelial biomass, and concentration of contaminated proteins were used as indicators to evaluate the yield and purity of *Fo*-EVs. The optimal method for *Fo*-EV preparation and extraction was screened by comparing liquid culture, solid culture, and solid culture with enzymatic cell wall hydrolysis. The optimal system for *Fo*-EVs separation and purification was screened by a pairwise combination of three primary methods (Ultracentrifugation (UC), Ultrafiltration (UF), and Polyethylene glycol precipitation method (PEG)) and two secondary methods (Size-exclusion chromatography (SEC) and Aqueous two-phase system (ATPS)), respectively. The protein composition was identified via mass spectrometry technology, followed by GO annotation and GO enrichment analysis using whole-genome proteins as the background. Based on these steps, a *Fo*-EV protein library was constructed to reveal *Fo*-EV’s most active biological functions. The results showed that solid culture combined with the UC-SEC method could effectively enrich *Fo*-EVs with a typical cup-shaped membrane structure. The obtained *Fo*-EVs had an average particle size of 253.50 nm, a main peak value of 200.60 nm, a particle concentration of 2.04 × 10^10^ particles/mL, and a particle number per unit protein of 1.09 × 10^8^ particles/μg, which were significantly superior to those of other combined methods. Through proteomic analysis, 1931 proteins enriched in *Fo*-EVs were identified, among which 350 contained signal peptides and 375 had transmembrane domains. GO enrichment analysis revealed that these proteins were mainly involved in cell wall synthesis, vesicle transport, and pathogenicity-related metabolic pathways. Additionally, 9 potential fungal EV markers, including Hsp70, Rho GTPase family, and SNARE proteins, were screened. This study constructed an isolation system and a marker database for *Fo*-EVs, providing a methodological and theoretical basis for in-depth analysis of the biological functions of *Fo*-EVs.

## 1. Introduction

Filamentous fungi are the most common plant pathogens, causing annual losses in yield and quality in agricultural and horticultural production [1,2]. During the process of infecting plants, these pathogens secrete a series of complex molecules, including toxins, plant cell wall-degrading enzymes, and effector proteins. These molecules reduce the host’s defense mechanisms and support the growth of the pathogens, thereby facilitating the pathogens’ colonization in the host tissues [3,4,5]. Among them, *Fusarium oxysporum* is a widely distributed soil-borne pathogenic fungus. The vascular bundle diseases caused by it can lead to severe yield reduction or even total crop failure of various cash crops and medicinal plants, resulting in significant economic losses [6,7]. In the research of pathogenetic fungus–host plant interaction, fungal extracellular vesicles (EVs) have attracted considerable attention due to their characteristic of carrying bioactive molecules [8,9]. However, the current isolation and analysis techniques for fungal EVs are mainly adapted from the research system of mammalian EVs. Fungal EVs still lack universal biomarkers that have been widely validated in mammalian systems, such as the tetraspanin protein CD63 [10,11]. This limitation significantly restricts the specific identification of fungal EVs [12].

The isolation of fungal EVs was first conducted on *Alternaria infectoria*, a pathogen that causes crop leaf spot disease [13]. Later, EVs from fungi, such as *Zymoseptoria tritici*, *F. graminearum*, and *F. oxysporum* were isolated by subjecting fungal liquid cultures to high-speed centrifugation [14,15,16]. However, the biofluid components of liquid cultures are highly complex, making it difficult to isolate and purify EVs from them using a single method. This significantly undermines the downstream analysis and application of EVs [17]. Although scholars have successively used synthetic culture media with fewer nutrients for liquid culture of fungi to reduce the interference of culture media and fungal metabolites in the subsequent isolation of EVs, the co-purification issue associated with single isolation methods remains unresolved [18].

The isolation methods for fungal EVs mainly include ultracentrifugation (UC), density gradient centrifugation, polymer precipitation, ultrafiltration (UF), and size-exclusion chromatography (SEC); however, each of these methods has its own limitations. UC is the most classic method for isolating EVs and is applicable to most EV isolation scenarios. However, the EVs obtained through this method are often co-precipitated with non-vesicular particles and protein aggregates, resulting in severe contamination [19,20,21]. Density gradient centrifugation can harvest high-purity EV subpopulations based on their respective densities, but the excessively long separation time and extremely high centrifugal speed often cause the rupture of the EV bilayer membrane, leading to a significant reduction in their biological activity [22]. UF is simple to operate and has limited equipment requirements. However, it is ineffective for processing samples with extremely large volumes or high levels of contaminating proteins; meanwhile, it also suffers from a relatively high risk of protein contamination [23]. SEC enables the isolation of EVs based on their size and can effectively remove protein contaminants, but it is not suitable for processing large-volume samples and requires the establishment of an appropriate separation system prior to isolation [24]. Some polymer-based methods for isolating EVs, such as polymer sedimentation and the Aqueous Two-Phase System (ATPS), are easy to operate and have low equipment requirements. However, due to the inherent properties of polymers, they tend to exacerbate protein polymer contamination in the final product, which, in turn, affects the downstream analysis of EVs [23]. Immunocapture-based methods, such as immunomagnetic beads and flow cytometry for isolating EVs, typically yield EV subpopulations with the highest purity. However, in fungal EV research, the lack of essential marker proteins makes these methods inapplicable [25].

There are significant physicochemical differences between EVs, biomacromolecules, and other subpopulations, which leads to the widespread issue that existing isolation methods selectively enrich specific EV populations [26,27]. Additionally, current biomarkers for fungal EVs are mostly derived from proteomic or transcriptomic data of crudely extracted samples. The co-precipitated extracellular molecules mixed in these samples pose a major challenge to the analysis of the true composition and function of fungal EVs. Therefore, establishing an efficient and high-purity isolation system for fungal EVs is crucial for in-depth studies on the key functions of EVs (such as immune regulation and virulence transmission) during the infection process of pathogenic fungi.

To this end, this study aims to screen and establish an optimized EV preparation protocol for *Fo* by systematically comparing multiple isolation strategies. This protocol will lay a solid foundation for subsequent accurate proteomic analysis and functional research, thereby deepening understanding of the biological mechanisms of fungal EVs.

## 2. Materials and Methods

### 2.1. Strain Preservation and Cultivation

The test strain *F. oxysporum* was isolated, identified and preserved by our laboratory from the diseased plants of *Fritillaria thunbergia* Miq. affected by root rot [28]. After *F. oxysporum* was cultured on PDA medium for 3 days, a fungal disc with a diameter of 5 mm was taken from the edge of the mycelium growth using a sterile punch. The fungal disc was inoculated into 200 mL of potato dextrose broth (PDB) and cultured at 25 °C with a rotation speed of 120 rpm for 5 days. The culture broth was filtered through four layers of sterile gauze to remove the mycelium, then centrifuged at 4000 r/min for 20 min to precipitate the spores. The spores were washed three times with sterile PBS, and the number of spores was counted using a hemocytometer. The spore suspension was resuspended in 25% glycerol, diluted to 1 × 10^6^ spores/mL, and stored at −80 °C for later use.

### 2.2. Selection of Cultivation and Pretreatment Methods

*F. oxysporum* was cultured and pretreated using three methods: solid culture, liquid culture, and solid culture followed by enzymatic hydrolysis. The optimal culture and treatment method was screened by separately characterizing the EVs isolated from each approach. For liquid culture, 100 μL of *F. oxysporum* spore suspension (1 × 10^6^ spores/mL) was inoculated into 200 mL of PDB medium, followed by incubation at 25 °C in the dark with shaking at 120 rpm for 3 days. For solid culture, a fungal disc with a diameter of 5 mm was inoculated onto the center of PDA covered with cellophane, followed by incubation at 25 °C in the dark for 3 days [29], harvesting the mycelium, immersing it in 100 mL of pre-chilled PBS-EDTA (10 mM PBS, 5 mM ethylenediaminetetraacetic acid) solution, and incubating at 80 rpm and 4 °C for 8 h. The culture method of solid culture combined with enzymatic hydrolysis is the same as that of the solid culture method. The harvested mycelia were immersed in 100 mL of enzymatic hydrolysate (0.7 M NaCl, 10 mM PBS, 100 mg/mL amylase (Novozymes Biotechnology, Tianjin, China), pH 5.5), and incubated at 80 rpm and 37 °C for 1 h [30]. For each method, three biological replicates were established. In the case of the solid culture method, each independent replicate consisted of a batch of 20 Petri dishes.

### 2.3. Acquisition of Fo-EVs

Mycelia and lysates from each of the above treatments were removed by sterile gauze filtration, and the supernatant was collected. The supernatant was subjected to gradient centrifugation at 4 °C sequentially as follows: 3500× *g* for 20 min, 7500× *g* for 20 min, and 11,000× *g* for 60 min. The supernatant was then filtered through a 0.22 µm sterile syringe filter to obtain the crude *Fo*-EV isolate. The crude *Fo*-EV isolate was centrifuged at 100,000× *g* and 4 °C for 70 min using an Optima XPN-100 ultra-high-speed refrigerated centrifuge (Beckman Coulter, Brea, CA, USA) to pellet the vesicles. The pellets were combined, resuspended, and centrifuged again. Finally, all pellets were resuspended in 300 μL of PBS to obtain the concentrated *Fo*-EV solution.

### 2.4. Methodological Research on the Separation and Purification of Fo-EVs

A methodological study on the isolation and purification of *Fo*-EVs was conducted by combining UC, UF, and PEG precipitation methods with SEC and ATPS, using UC, UF, and PEG as control groups.

The procedure for UC was the same as described in Section 2.3. For UF, the initial separation solution was concentrated to 300 μL using an ultrafiltration tube (Amicon Ultra-15, 100 kDa, Merck Millipore, Darmstadt, Germany) under the conditions of 4000× *g*, 4 °C for 15 min, and the *Fo*-EV concentrate was collected. For the PEG precipitation method, the initial separation solution was thoroughly mixed with a PEG precipitation solution (30% PEG8000, 1.5 M NaCl) to achieve a final concentration of 10% for PEG8000 [31]. After incubation at 4 °C overnight, centrifugation was performed at 10,000× *g*, 4 °C for 60 min. The collected precipitate was resuspended in 300 μL of PBS, and the *Fo*-EV concentrate was harvested.

SEC involves mixing 300 μL of *Fo*-EV concentrate with 5 μL of Dio fluorescent probe (Lanjieke Technology, Beijing, China) to achieve a final Dio concentration of 10 μM. After incubation at 37 °C for 1 h in the dark, the mixture was purified using a Sepharose CL-2B [17] exclusion column (Yuanye Biotechnology, Shanghai, China). The exclusion column was equilibrated and eluted with PBS-EDTA at a flow rate of 1 mL/min. The eluate was collected in black microtiter plates at 300 μL per fraction under dark conditions. The fluorescence intensity and concentration of contaminating proteins in each fraction were determined, with three biological replicates set up. A coordinate system was established with the separated fractions in chronological order as the abscissa, and the fluorescence intensity and protein content of the fractions as the ordinate. Ten consecutive fractions rich in vesicles with low levels of contaminating proteins were identified as the standard for subsequent SEC separation and purification. ATPS [32] involves thoroughly mixing 300 μL of *Fo*-EV concentrate with an equal volume of 2× ATPS separation solution (2.60% dextran, *w*/*w*; 9% polyethylene glycol 6000, *w*/*w*; 1% Coomassie Brilliant Blue R250, *v*/*v*). After the mixture was allowed to stand at room temperature for 10 min, it was centrifuged at 1000× *g* for 10 min. After that, 80% of the upper phase liquid was removed and replaced with the upper phase of the washing solution, followed by inversion and mixing in the same manner. After standing and centrifuging again, the upper polyethylene glycol phase was completely removed. Approximately 10% of the total volume of the *Fo*-EV solution was collected from the dextran layer, and the volume was adjusted to 300 μL with PBS solution.

### 2.5. Characterization of Fo-EVs

The particle size distribution and concentration were measured using a Nanoparticle Tracking Analyzer (NTA) NanoSight NS500 (Malvern Instruments, Malvern, UK). Samples were diluted 1000-fold with PBS to ensure the number of particles per frame ranged from 30 to 200. The software settings were as follows: camera level of 15, detection threshold of 10, viscosity of 0.794–0.797 centipoise (cP), detection temperature of 25 °C, acquisition duration set to 60 s, and a total of 5 technical replicates were performed.

Transmission Electron Microscopy (TEM) observation was conducted using a Hitachi H-7650 microscope (Hitachi, Tokyo, Japan). A 10 μL aliquot of the sample was loaded onto a 200-mesh copper grid, which was then left to stand at room temperature for 1 min. The sample was negatively stained with 1% (*w*/*v*) uranyl acetate for 1 min; after drying, images of the sample were captured at an acceleration voltage of 80 kilovolts (kV).

The protein content was determined using a micro-Bicinchoninic Acid (BCA) assay kit. A standard curve was generated using purified Bovine Serum Albumin (BSA). The absorbance of the samples was measured at a wavelength of 595 nanometers (nm) using a microplate reader, and the protein content of the *Fo*-EV samples was calculated and determined accordingly.

The number of particles per unit mycelial biomass was measured. Mycelia from solid culture and liquid culture were collected, placed in a Petri dish, and dried in an oven at 40 °C until their mass remained constant. Each group included 5 biological replicates. The mycelial biomass harvested from the two culture methods was calculated by weighing, and the number of particles per unit mycelial mass was finally computed based on the particle concentration measured via NTA.

### 2.6. Protein Preparation and Silver Staining

*Fo*-EV samples were isolated and purified using a combination of the solid-state culture method and UC-SEC. For each isolation experiment, fungal cultures from 40 Petri dishes were used as the starting material, with a total of three biological replicates set up. For mycelial samples, mycelia were also obtained via the solid-state culture method. After quick-freezing in liquid nitrogen, the mycelia were ground using a grinder (Jingxin Industrial Development, Shanghai, China) under the conditions of 60 Hz for 2 min. Both *Fo*-EVs and mycelial samples were lysed with RIPA strong lysis buffer (Biyun Tian Biotechnology, Shanghai, China). After incubation on ice for 20 min, the mixtures were centrifuged at 12,000× *g* and 4 °C for 5 min, and the supernatants were collected. *Fo*-EV proteins and mycelial proteins were concentrated using 3 kDa ultrafiltration tubes. The protein concentration was determined by the BCA method and adjusted to ensure that the loading amount of *Fo*-EV protein per lane in electrophoresis was approximately 10 µg. SDS-polyacrylamide gels (10% separating gel, 5% stacking gel) were prepared for protein electrophoresis. The electrophoresis was performed at a constant voltage of 80 V for 40 min; when the bromophenol blue band migrated to 1 cm from the bottom of the gel, the voltage was switched to a constant 120 V. After electrophoresis, the gel was stained using a rapid silver staining kit (Sangon Biotech, Shanghai, China).

### 2.7. Mass Spectrometry

Mass spectrometry analysis of EV protein samples was conducted by Guangke Ande Co., Ltd. (Hangzhou, China). Mass spectrometric identification was performed using the Data-Independent Acquisition (DIA) strategy. After desalting and lyophilization, the peptides were separated on the Vanquish Neo Nano LC System (Thermo Fisher Scientific, Shanghai, China). Chromatographic separation was carried out on a High Throughput μPAC Neo HPLC Column (length: 5.5 cm, inner diameter: 75 μm) with the column temperature maintained at 55 °C. Mobile phase A consisted of 0.1% formic acid, 2% acetonitrile, and 98% ultrapure water, while mobile phase B was composed of 0.1% formic acid, 80% acetonitrile, and 20% ultrapure water. The flow rate was set at 2 μL/min. The gradient elution program was as follows: 4–12% B (0–0.2 min), 12–25% B (0.2–3.2 min), 25–50% B (3.2–5.8 min), 50–99% B (5.8–6.2 min), and 99% B (6.2–6.9 min). The sample injection volume was 200 ng.

Following chromatographic separation, the peptides were directly introduced into the Astral High-Resolution Mass Spectrometer (Thermo Fisher Scientific, Shanghai, China) for DIA detection. Mass spectrometric data were acquired in positive ion mode with a spray voltage of 2000 V and an ion transfer tube temperature of 290 °C. For the full-scan mass spectrometry (MS1), the scan range was set at 380–980 *m*/*z*, the resolution was 240,000 (at 200 *m*/*z*), the automatic gain control (AGC) target was 5 × 10^6^, and the maximum injection time was 5 ms. For the tandem mass spectrometry (MS2/DIA), data acquisition was performed with 299 isolation windows (window width: 2 *m*/*z*). The collision energy was set at 25 eV (higher-energy collisional dissociation, HCD), the AGC target was 5 × 10^6^, the maximum injection time was 3 ms, and the MS2 scan range covered 150–2000 *m*/*z*.

The raw data were subjected to database searching and quantitative analysis using DIA-NN software (v1.8.1). The key parameters for data processing were set as follows: trypsin was specified as the digestive enzyme with a maximum of 1 missed cleavage allowed; carbamidomethylation of cysteine was set as a fixed modification, while oxidation of methionine and acetylation of protein N-termini were defined as variable modifications; the false discovery rate (FDR) threshold for protein identification was set at 1%. Data quality control was primarily evaluated based on the following indicators: peptide mass error (ppm), length distribution of trypsin-digested peptides, and Pearson Correlation Coefficient (PCC) among biological replicate samples.

### 2.8. Fo-EV Protein Functional Annotation and Enrichment Analysis

Protein screening was performed based on the maximum abundance change ratio between replicate experiments ≤ 1.2 and the presence of two or more unique peptide segments. The protein accession numbers of the screened *Fo*-EV proteins were queried using Uniprot to identify the presence of proteins containing transmembrane domains (TMD) and signal peptides (SP). The eggONG tool was used to perform Gene Ontology (GO) annotation analysis on proteins with and without TMDs. Proteins were classified into enzymes based on their enzyme commission (EC) numbers. Using the entire genome protein as a background, g: Profiler was used to perform GO enrichment analysis on the analyzed proteins (*p* < 0.001, FDR < 0.05).

## 3. Results

### 3.1. The Effect of Cultivation and Pretreatment Methods on the Separation Efficiency of Fo-EVs

**Figure 1:**
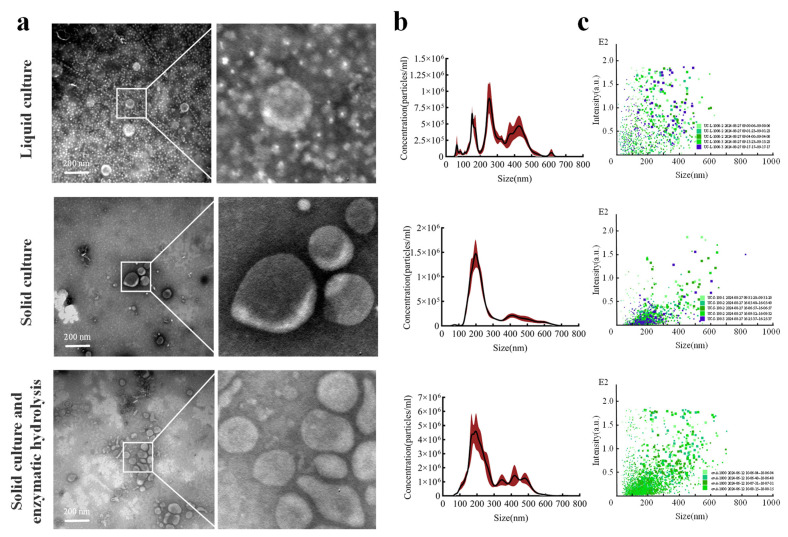
Effect of culture method and enzymatic hydrolysis on *Fo*-EV yield: (**a**) electron microscopy characterization; (**b**) particle size distribution (red section indicates standard error); (**c**) signal intensity of particle size distribution.

*Fo*-EVs derived from liquid culture, solid culture, and solid culture with enzymatic hydrolysis were successfully isolated using the UC method. All centrifugal precipitates appeared dark purple. TEM images (Figure 1a) revealed that the diameter of *Fo*-EVs was approximately 200 nm, and they exhibited the characteristic cup-shaped closed membrane structure unique to EVs. Compared with the simple solid culture group, the liquid culture group and the solid culture with enzymatic hydrolysis group showed more complex backgrounds under TEM; aggregated heteroproteins masked some of the vesicles. Analysis of the particle size distribution (Figure 1b) and particle signal intensity distribution (Figure 1c) of *Fo*-EVs provided the following insights: In the solid culture group, the particle size and signal intensity distributions were concentrated, with a single prominent peak around 200 nm. Particles of all sizes were concentrated in the signal intensity range of 0–0.5, indicating a relatively homogeneous particle composition. The liquid culture group contained three types of particles with different sizes, and their signal intensities were scattered (particles of various sizes were randomly distributed across the signal intensity range of 0–2). This may be due to the presence of insoluble protein polymers or other impurity particles (impurities such as protein polymers exhibit relatively high signal intensities), so the actual number of particles in the liquid culture should be lower than the measured data. The solid culture with enzymatic hydrolysis group had more particles in the range of 300–500 nm, and the number of particles with high signal intensity increased (Figure 1c). This could be attributed to the enzymatic hydrolysis treatment releasing more contaminating proteins (1.87 mg/mL), which caused protein aggregation and resulted in the detection of more large particles [33]. Table 1 provides the details of Particle concentration, Mean, Mode, Number of protein particles per unit, Number of biomass particles per unit, and Contaminant protein concentration.

### 3.2. Establishment of a Size Exclusion Separation System for Fo-EVs

**Figure 2:**
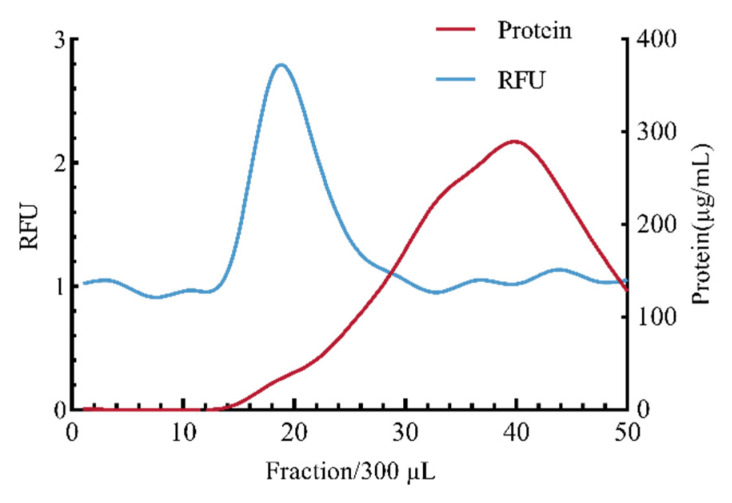
Establishment of *Fo*-EV size exclusion purification system (*n* = 3).

As shown in Figure 2, the elution fraction range for size exclusion separation of *Fo*-EVs was determined based on the relative fluorescence intensity (RFU) and protein concentration at each fraction. Fluorescence was primarily concentrated in fractions 17–24, while eluted impurity proteins gradually increased starting from fraction 14, peaking at fraction 40. To minimize operational errors in the separation experiment, elution fractions 16–25 were selected as the standard for subsequent size exclusion chromatography experiments.

### 3.3. The Effect of Separation Methods on the Separation Efficiency of Fo-EVs

**Figure 3:**
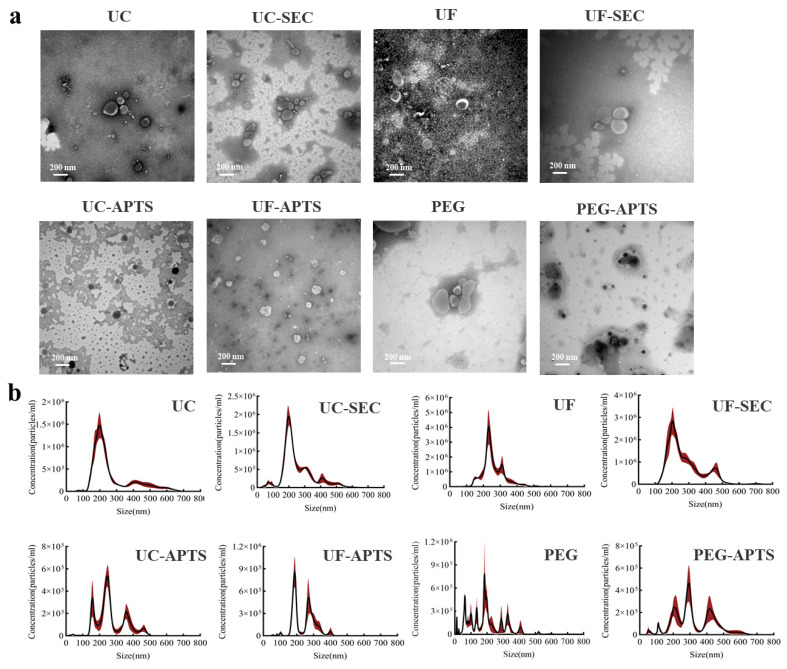
Effect of different separation methods on the separation efficiency of *Fo*-EVs: (**a**) electron microscope characterization of different separation methods; (**b**) particle size distribution of different separation methods (red shaded area indicates standard error).

TEM characterization (Figure 3a) showed that vesicular structures were present in all combined isolation methods. Among these methods, UC, UF, UC-SEC, UF-SEC and PEG exhibit the typical cup-shaped bilayer membrane structure of EVs, while the three methods—UC-ATPS, UF-ATPS, and PEG-ATPS—show a complex background and blurry vesicle edges. From the perspective of particle size distribution (Figure 3b), there was almost no difference in the *Fo*-EVs isolated by UC, UC-SEC, and UF-SEC. All had a mode of 200 nm, a mean of approximately 260 nm, and a concentrated particle distribution. In contrast, the *Fo*-EVs isolated by the four methods of UC-ATPS, UF-ATPS, PEG and PEG-ATPS exhibited a wide particle size distribution (150 nm–500 nm) with multiple signal peaks corresponding to different particle sizes. Table 2 summarizes the physical characteristics of the separated *Fo*-EVs, including the particle concentration, Mean, Mode, and the number of protein particles per unit. For the PEG-SEC method, the presence of polymers led to excessively slow eluent flow rate and severe protein aggregation during SEC separation. Therefore, this method is no longer considered for the establishment of the isolation system.

### 3.4. Protein Electrophoresis Silver Staining

**Figure 4:**
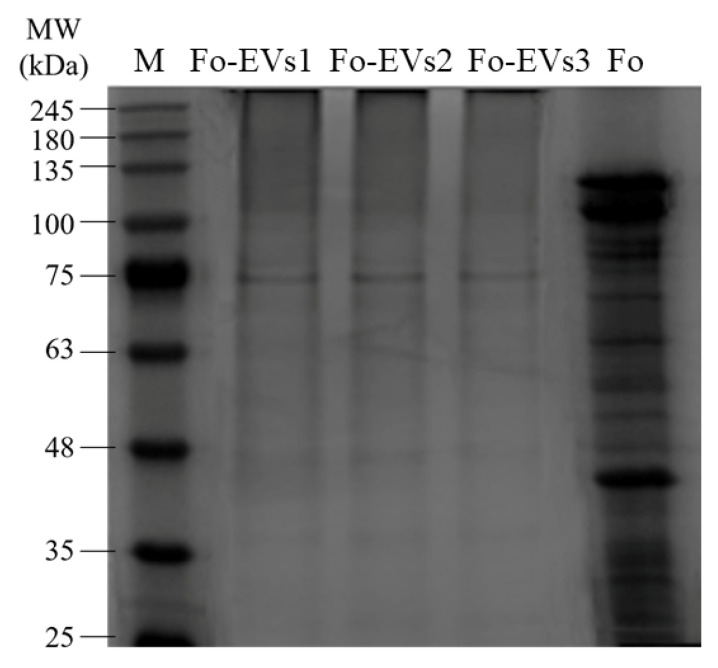
SDS-PAGE electrophoresis silver staining results of *Fo*-EV protein and mycelium protein *Fo*-EVs1, *Fo*-EVs2, and *Fo*-EVs3 represent three biological replicates of *Fo*-EVs purified using the solid culture method combined with UC-SEC. *Fo* represents the total protein from *Fo* mycelia.

Silver staining analysis revealed that *Fo*-EVs have a specific protein composition (Figure 4). The most intensely stained band was observed at approximately 75 kDa, indicating the presence of one or more highly abundant proteins at this position. Compared with the total protein profile of *Fo* mycelia, the protein profile of *Fo*-EVs showed significant differences. Specifically, several protein bands in the 100–135 kDa range and a distinct band at 40 kDa in the mycelial protein profile were either completely absent or significantly reduced in intensity in the *Fo*-EV protein profile.

### 3.5. Fo-EVs GO Annotation

We performed mass spectrometry analysis on three biological replicates of *Fo*-EVs (*Fo*-EVs1, *Fo*-EVs2, and *Fo*-EVs3) purified by the solid culture method combined with UC-SEC. The results showed good data quality, as well as high reproducibility and good consistency among the samples. A total of 2790 proteins were identified through the mass spectrometry analysis of *Fo*-EV proteins. Based on the criteria that the maximum abundance change ratio between duplicate experiments was ≤1.2 and the presence of 2 or more unique peptides, 1931 specific EV proteins were screened out. Among them, only 350 proteins contained SP, indicating that EVs were well separated from the free proteins secreted by fungi through the conventional secretory pathway. The 1931 identified proteins were divided into 375 membrane-bound proteins containing TMD and 1556 water-soluble proteins, which were subjected to annotation analysis, respectively. This classification, focusing on membrane association rather than secretory signals, was chosen to reflect the final functional assembly of the EV proteome, which incorporates proteins from multiple pathways.

**Figure 5:**
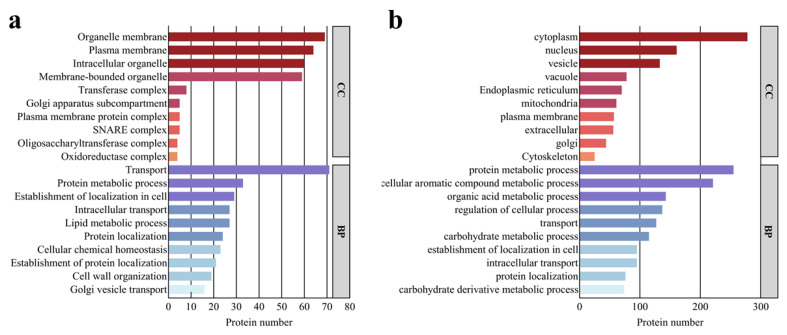
GO annotation of proteins in *Fo*-EVs: (**a**) GO annotation of proteins containing TMD in *Fo*-EVs; (**b**) GO annotation of proteins not containing TMD in *Fo*-EVs.

In the GO analysis of the Biological Process (BP) category for the 375 proteins containing TMD, the top 10 annotations mainly highlighted transport, establishment of localization, protein and lipid metabolism, maintenance of cellular homeostasis, and cell wall organization. The predicted subcellular localization of these proteins was primarily associated with the plasma membrane, endoplasmic reticulum, vacuoles, Golgi apparatus, and vesicles (Figure 5a), which implies the origin of vesicles and their involvement in the secretory pathway. Based on the Enzyme Commission (EC) numbers obtained from the KEGG analysis of these TMD-containing proteins, 85 of them were predicted to be enzymes, including: Oxidoreductases, Transferases (catalyzing the transfer of functional groups such as acyl, glycosyl, and phosphate groups), Hydrolases (catalyzing the hydrolysis of substances like phosphates, β-glucosides, and cellulose), Lyases (comprising ammonia lyases and nucleotide cyclase’s), Isomerases (RNA pseudouridine synthases), Translocases (ABC transporters and H^+^ transmembrane transporters). For the other 1556 proteins, their annotations mainly highlight the metabolism of organic substances and macromolecules (including proteins, lipids, aromatic compounds, and organic acids), as well as transport, establishment of cellular localization, and regulation of cellular processes. The predicted localization of these proteins is primarily associated with the cytoplasm, nucleus, vacuoles, vesicles, endoplasmic reticulum, mitochondria, and Golgi apparatus (Figure 5b). Based on the EC numbers, 822 of these proteins can be classified into six major enzyme categories: hydrolases, oxidoreductases, ligases, lyases, isomerases, and translocases. These results indicate that vesicles may serve as a crucial tool for *F. oxysporum* to adapt to low water activity environments, as they enable the fungus to utilize nutrients and maintain its own growth and development.

Among all the proteins analyzed, we identified multiple categories of potentially conserved fungal EV marker proteins based on the principles of homology, functional relevance, and cross-species conservation. Specifically, proteins including Hsp70, t-SNARE YKT6, GTPases Rho1 and Rho3, Sur7, and Vac8 were included [34] due to their high homology with the established list of Candida albicans EV marker proteins. Meanwhile, based on functional relevance, we confirmed 5 heat shock proteins, the ESCRT protein HSE1, various SNARE proteins, and one Sec protein—all of which are directly involved in key biological processes of EVs. Additionally, based on cross-species conservation, the Flotillin protein, which is used as an EV marker in mammalian systems [35], was also detected in the identification results of this study.

Fungal EVs are believed to play a role in cell wall synthesis and remodeling [36]. Among all the EV proteins, a total of 66 enzymes potentially involved in fungal cell wall remodeling were detected. These enzymes are mainly associated with the synthesis and metabolism of β-glucan, chitin, mannan, xylan, pectin, and chitosan. The presence of these proteins is consistent with a biological role of *Fo*-EVs in fungal cell wall synthesis and remodeling.

*F. oxysporum* enhances its virulence by secreting a variety of secondary metabolites. Among the detected *Fo*-EV proteins, several proteins involved in the synthesis of polyketides, steroids, and terpenoids were identified, including polyketide synthase X0MH33, farnesyl pyrophosphate synthase X0MTQ2, and taxadiene pyrophosphate synthase X0NJI9, as well as multiple proteins involved in other secondary metabolic pathways. Taxadiene pyrophosphate synthase is involved in the biosynthesis of toxins such as higginsianin A~E. These terpenoid α-pyrone compounds exhibit cytotoxicity to mammalian cells; in plants, higginsianin B can also inhibit the activation of the jasmonic acid defense signaling pathway [37]. In addition, polyketide synthases are directly responsible for the synthesis of important Fusarium toxins, such as deoxynivalenol (vomitoxin) and zearalenone [38].

### 3.6. BP Enrichment Analysis of Fo-EV Proteins

**Figure 6:**
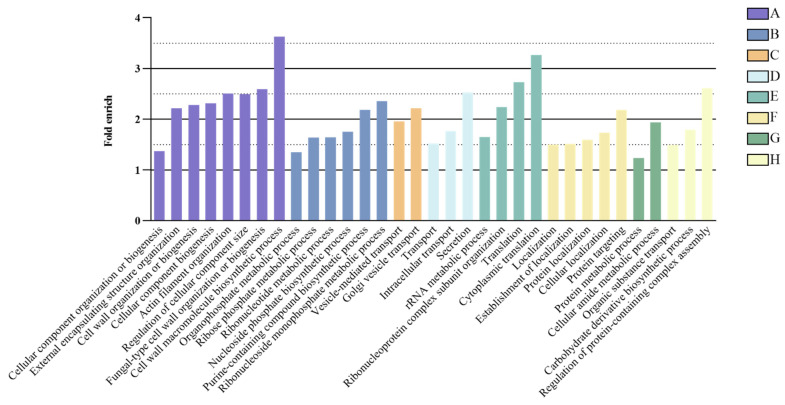
GO enrichment analysis of proteins in *Fo*-EVs: (A. cell components biogenesis and organization, including cell wall and cytoskeleton metabolism; B. nucleotide and metabolite biosynthesis; C. vesicle transport; D. membrane transport; E. ribosomes and translation; F. localization; G. nitrogen metabolism and protein metabolism; H. metabolic regulation and signal transduction).

Using the whole-genome protein set of *F. oxysporum* as the background, GO enrichment analysis was performed on the identified *Fo*-EV proteins, followed by manual curation (Figure 6). The 1931 *Fo*-EV proteins could be classified into 8 major related categories: Biogenesis and organization of cellular components (including metabolism of cell wall and cytoskeleton), Biosynthesis of nucleotides and metabolites, vesicular transport, membrane transport, ribosome and translation, localization, nitrogen metabolism and protein metabolism, metabolic regulation and signal transduction. The abundant presence of these proteins in *Fo*-EVs reflects that *Fo*-EVs play important roles in plasma membrane and cell wall synthesis, pathogenesis, stress response, transport, signal transduction, and basic cellular metabolism.

## 4. Discussion

This study compared the effects of different culture methods and pretreatment methods on the isolation of *Fo*-EVs. The results showed that the methods of isolating *Fo*-EVs using liquid culture and enzymatic hydrolysis pretreatment yielded a higher number of particles, but also resulted in more contaminating proteins. This finding is consistent with the complex background noise observed in TEM characterization, as well as the broad particle size and signal intensity distribution detected in NTA. This phenomenon may be attributed to the following reasons: compared with solid culture, *F. oxysporum* exhibits more active metabolic processes in liquid culture, and the components of the medium used for liquid culture increase the complexity of the starting material for *Fo*-EV isolation. For enzymatic hydrolysis pretreatment, it releases a large number of vesicles present in the region between the fungal cell wall and plasma membrane; meanwhile, the oscillation during enzymatic hydrolysis causes mechanical damage to the vesicles, leading to the release of their contents. Under natural conditions, plant-pathogenic filamentous fungi mostly grow on solid substrates [39], and the quantity of EVs extracted from solid culture is sufficient for downstream analyses [31]. Therefore, after careful consideration, the culture method using cellophane to separate fungal mycelia from the solid medium is deemed a suitable choice for fungal EV research.

Before the discovery of universal marker proteins for fungal EVs, it was an inevitable approach to isolate and purify EVs based on their physicochemical properties. Therefore, in this study, through the comparative evaluation of various isolation methods, it was found that the UC-SEC method is the most suitable for the subsequent experiments of this study. It not only exhibits favorable electron microscopy characterization, particle concentration, and particle size distribution but also achieves relatively high purity. However, the other methods also possess unique advantages in other aspects. The UF method and PEG precipitation method have simple equipment requirements and convenient operation. They can also ensure the extraction and isolation of biomacromolecules from vesicles for subsequent studies, making them suitable for practical production and application scenarios. Both the SEC method and ATPS method can remove part of the contaminating proteins from *Fo*-EV samples, but unfortunately, they have polymer residues. The aqueous two-phase affinity method impairs the integrity of purified vesicles, and the residual dextran polymer affects downstream characterization. Nevertheless, the ATPS method enables the removal of contaminating proteins and requires only 30 min of operation time, which allows its application in some clinical diagnostic scenarios [40].

Based on current research, scholars generally hypothesize that fungal EVs are important tools for transporting virulence factors that were overlooked in the early days, and they constitute an indispensable part of fungal pathogenicity [36]. Among the biomolecules carried by EVs, proteins are a crucial component [41]. In this study, a series of proteins ranging in size from 35 to 245 kDa was isolated from *Fo*-EVs, and 2790 types of *Fo*-EV proteins were identified using mass spectrometry. A total of 1931 *Fo*-EV proteins were screened out based on the characteristic of small changes in EV protein abundance among biological replicates, and the number of identified protein types is much higher than that in similar EV studies. Compared with other studies, the most significant difference in this experiment lies in the use of *F. oxysporum* cultured in solid medium rather than liquid medium as the research object. Due to the low water content in solid culture, the utilization of nutrients is restricted; therefore, fungi need to secrete enzymes with higher concentrations and more diverse types to obtain essential nutrients. This viewpoint is supported by the presence of various proteases (such as amidases, hydrolases, peptidases, sulfatases, and aspartases) in *Fo*-EV proteins, which confirms that *Fo*-EVs can decompose and absorb complex biomolecules in an aseptic environment, thereby promoting the growth and development of *F. oxysporum*. The presence of such proteins has also been reported in studies on EVs released by several pathogenic yeast strains [42]. In addition, a study on *Botrytis cinerea* EVs using the same culture method as this study identified 2461 proteins from EVs [43], and a proteomic study on *Aspergillus oryzae* under submerged-mixed and solid-state culture conditions [44] both confirm that the variety of proteins secreted in solid culture is far greater than that in submerged culture.

Compared with cells or organelles with larger diameters, EVs contain a higher proportion of phospholipid bilayers; therefore, plasma membrane-associated proteins often show high-abundance expression in EVs. Accordingly, the International Society for Extracellular Vesicles (ISEV) has proposed that the two main categories of proteins for EV analysis are membrane-bound proteins and cytoplasmic proteins [45]. In this study, GO annotation was performed on the 375 screened membrane-bound proteins. It was found that their predicted localizations are associated with various cellular compartments, including the cell membrane, endoplasmic reticulum, vacuoles, and Golgi apparatus, which reflects the biogenesis process of *Fo*-EVs. Among these membrane-bound proteins, the SNARE protein YKT6 and the Sur7 protein are particularly noteworthy: they are highly promising EV marker proteins and have been detected in a variety of fungal EVs [43]. In the cytoplasmic proteins of *Fo*-EVs, potential candidates for fungal EV markers were also identified, including the heat shock protein Hsp70, GTPases Rho1 and Rho3, vacuolar protein Vac8, ESCRT protein HSE1, and SEC22 protein. These proteins have all been reported in studies on *Candida albicans* [34], *Colletotrichum higginsianum* [30], and other fungi. Different from other EV studies, this study also identified the Flotillin protein. It is a highly conserved transmembrane-associated protein that is widely present in the cells of various organisms and mainly associated with membrane microdomains. It may also serve as one of the EV marker proteins, similar to the EV markers Flotillin-1 and Flotillin-2 in mammalian systems [46].

In numerous research reports on fungal EVs, cytoplasmic proteins are present in extremely high abundance in EVs. Thus, the EV proteome reflects the cytoplasmic protein content of cells to a certain extent. However, most studies focusing on the protein content of fungal EVs have not investigated the relative abundance of EV proteins in whole-cell lysates, even though this investigation would actually contribute to exploring the functions and biomarkers of fungal EVs. In the present study, GO enrichment analysis was performed on *Fo*-EV proteins, with the whole-genome protein set of *F. oxysporum* used as the enrichment background. The results showed that *Fo*-EV proteins were mainly enriched in processes including cell wall biogenesis, plasma membrane-related functions, stress response, pathogenesis, membrane transport, and signal transduction. Additionally, a relatively high proportion of these proteins was associated with basic cellular metabolic functions, such as the synthesis and degradation of proteins and carbohydrates. These findings reflect that *Fo*-EVs are closely related to the maintenance of fungal cell walls and biofilms, fungal immunostimulatory activity, substance transport, and virulence. Furthermore, some proteins associated with the cytoskeleton and cell wall are believed to play a role in the process of fungal EVs passing through the cell wall.

## 5. Conclusions

This study successfully established a *Fo*-EV separation and purification system using solid culture combined with UC-SEC. The *Fo*-EVs isolated by this method exhibited typical vesicular morphological characteristics, with a particle concentration of 2.04 × 10^10^ particles/mL, an average particle size of 253.50 nm, and a peak particle size of 200.60 nm. Additionally, they had a low content of contaminating proteins (2.13 mg/mL), and the number of particles per unit protein reached 1.09 × 10^8^ particles/μg. UC-SEC separates based on the general physical properties of vesicles, and this method can be extended to the study of other fungal EVs. In the absence of specific marker proteins for fungal EVs, the establishment of a standardized isolation method helps to standardize the quality of EV samples across different studies and provides a comparable basis for downstream nucleic acid and protein analyses, thereby advancing progress in the research field of fungal EVs.

Through proteomics analysis, we identified nine potential EV marker proteins in *Fo*-EVs, including the heat shock protein Hsp70, GTPase Rho1/Rho3, vacuolar protein Vac8, ESCRT protein HSE1, SEC22 protein, Flotillin protein, SNARE protein YKT6, and Sur7 protein. SDS-PAGE, Western blot, and mass spectrometry analysis indicated that the *Fo*-EVs proteome is closely associated with biological processes such as cell wall synthesis, fungal virulence, and immune stress. These findings not only deepen our understanding of plant–fungal pathogen interactions but also provide important evidence for the screening and functional studies of fungal EV marker proteins.

## Figures and Tables

**Table 1:** Effect of culture method and enzymatic hydrolysis on *Fo*-EV yield.

	Particle Concentration (Particles/mL)	Mean (nm)	Mode (nm)	Number of Protein Particles Per Unit (Per μg)	Number of Biomass Particles Per unit (Per gram)	Contaminant Protein Concentration (mg/mL)
liquid culture	1.40 × 10^11^ ± 8.25 × 10^9^	305.20	233.60	5.93 × 10^7^	2.37 × 10^10^	2.36
solid culture	2.82 × 10^10^ ± 7.83 × 10^9^	267.90	201.80	2.22 × 10^7^	8.48 × 10^9^	1.27
solid culture and enzymatic hydrolysis	7.73 × 10^11^ ± 3.41 × 10^10^	274.40	192.70	4.14 × 10^8^	2.32 × 10^11^	1.87

**Table 2:** Effect of separation methods on the separation efficiency of *Fo*-EVs.

	Particle Concentration (Particles/mL)	Mean (nm)	Mode (nm)	Number of Protein Particles Per Unit (Per μg)
UC	2.82 × 10^10^ ± 7.83 × 10^9^	267.90	201.80	2.22 × 10^7^
UC-ATPS	5.25 × 10^10^ ± 9.65 × 10^9^	272.20	223.50	5.11 × 10^7^
UC-SEC	2.04 × 10^10^ ± 8.25 × 10^8^	253.50	200.60	7.93 × 10^7^
UF	3.15 × 10^10^ ± 1.62 × 10^9^	258.10	245.20	1.40 × 10^7^
UF-ATPS	5.75 × 10^10^ ± 6.75 × 10^9^	237.26	225.28	4.30 × 10^7^
UF-SEC	3.87 × 10^10^ ± 3.23 × 10^9^	272.90	209.50	2.86 × 10^7^
PEG	5.78 × 10^10^ ± 5.93 × 10^9^	194.10	184.60	2.57 × 10^7^
PEG-ATPS	6.00 × 10^10^ ± 7.83 × 10^9^	323.00	256.10	1.17 × 10^8^

## Data Availability

The mass spectrometry proteomics data were deposited to ProteomeXchange Consortium via the PRIDE partner repository with the dataset identifier PXD071461.
